# Adequate antiviral treatment lowers overall complications of cytomegalovirus colitis among inpatients with inflammatory bowel diseases

**DOI:** 10.1186/s12879-024-09317-w

**Published:** 2024-04-26

**Authors:** Ching-Reigh Hsieh, Ren-Chin Wu, Chia-Jung Kuo, Pai-Jui Yeh, Yuan-Ming Yeh, Chyi-Liang Chen, Cheng-Tang Chiu, Cheng-Hsun Chiu, Yu-Bin Pan, Yung-Kuan Tsou, Puo-Hsien Le

**Affiliations:** 1 Department of Gastroenterology and Hepatology, Chang Gung Memorial Hospital, Linkou, Taoyuan, Taiwan; 2 Department of Anatomic Pathology, Chang Gung Memorial Hospital, Linkou, Taoyuan, Taiwan; 3 Taiwan Association of the Study of Small Intestinal Disease, Taoyuan, Taiwan; 4 Chang Gung Microbiota Therapy Center, Taoyuan, Taiwan; 5 Division of Pediatric Gastroenterology, Department of Pediatrics, Chang Gung Memorial Hospital, Linkou, Taoyuan, Taiwan; 6 Genomic Medicine Core Laboratory, Chang Gung Memorial Hospital, Linkou, Taoyuan, Taiwan; 7 Molecular Infectious Disease Research Center, Chang Gung Memorial Hospital, Linkou, Taoyuan, Taiwan; 8 Division of Pediatric Infectious Diseases, Department of Pediatrics, Chang Gung Memorial Hospital, Linkou, Taoyuan, Taiwan; 9 Biostatistical Section, Clinical Trial Center, Chang Gung Memorial Hospital, Linkou, Taoyuan, Taiwan; 10 Liver Research Center, Chang Gung Memorial Hospital, Linkou, Taoyuan, Taiwan; 11 Chang Gung Inflammatory Bowel Disease Center, Taoyuan, Taiwan

**Keywords:** Cytomegalovirus, Inflammatory bowel disease, Risk factor, Antiviral treatment, Complication

## Abstract

**Background:**

Cytomegalovirus (CMV) colitis significantly complicates the course of inflammatory bowel disease (IBD), frequently leading to severe flare-ups and poor outcomes. The role of antiviral therapy in hospitalized IBD patients with CMV colitis is currently under debate. This retrospective analysis seeks to clarify the influence of antiviral treatment on these patients.

**Methods:**

We retrospectively reviewed IBD patients diagnosed with CMV colitis via immunohistochemistry staining from colonic biopsies at a major tertiary center from January 2000 to May 2021. The study focused on patient demographics, clinical features, risk factors, prognostic indicators, and antiviral treatment outcomes.

**Results:**

Among 118 inpatients, 42 had CMV colitis. Risk factors included hypoalbuminemia and antibiotic use. IBD patients with CMV colitis receiving < 14 days of antiviral therapy had higher complication (72% vs. 43%, *p* = 0.028) and surgery rates (56% vs. 26%, *p* = 0.017) compared to those without CMV. Adequate antiviral therapy (≥ 14 days) significantly reduced complications in the CMV group (29% vs. 72%, *p* = 0.006), especially in Crohn’s disease (20% vs. 100%, *p* = 0.015). Independent predictors of IBD-related complications were CMV colitis (Odds Ratio [OR] 3.532, 90% Confidence Interval [CI] 1.012–12.331, *p* = 0.048), biological treatment failure (OR 4.953, 95% CI 1.91-12.842, *p* = 0.001), and adequate antiviral therapy (OR 0.108, 95% CI 0.023–0.512, *p* = 0.005).

**Conclusion:**

CMV colitis and a history of biological treatment failure increase complication risks in IBD patients. Adequate antiviral therapy significantly mitigates these risks, highlighting its importance in managing IBD patients with CMV colitis.

## Introduction

Cytomegalovirus (CMV) is a double-stranded DNA virus in the Herpesviridae family and is known to cause gastrointestinal tract diseases in both immunocompromised and immunocompetent individuals [1]. The prevalence of CMV colitis is significantly higher in patients with inflammatory bowel disease (IBD) than in those without IBD, especially ulcerative colitis (UC) [2]. The reported prevalence ranges between 10 and 30% in steroid-refractory acute severe colitis [3]. There is still some inconsistency in the literature regarding the role of CMV infection in exacerbating the severity of inflammation and adverse outcomes in IBD, as well as the response to treatment [4]. Some studies have suggested that CMV colitis is associated with worse outcomes in IBD, including steroid resistance, increased risk of colectomy, inpatient mortality, and longer hospital stays [5–9]. However, other studies have not found a significant association between CMV infection and these adverse outcomes in IBD [10, 11].

Additionally, the effectiveness of antiviral treatment for CMV colitis in IBD remains a topic of debate, with some studies reporting improved outcomes with antiviral treatment [12–16], while others have not found a significant benefit [10, 17]. Potential factors that could contribute to the inconsistent findings include differences in the study population, diagnostic tools, disease activity, treatment strategies, genetic predisposition, and viral load in the colonic tissue. Recently, it has been suggested that high-grade CMV disease, characterised by a high viral load (in serum and/or tissue), indicates that CMV acts as a pathogen and may exacerbate IBD severity. In contrast, low-grade CMV disease may reflect the influence of IBD itself on the outcome, rather than the direct contribution of CMV infection [4]. Accordingly, some published algorithms regarding the management of CMV infection in IBD patients suggested antiviral treatment in steroid refractory IBD patients with high-grade CMV disease [3, 14, 18]. High-grade CMV infections were diagnosed through the measurement of viral loads in both blood and tissue samples, with a particular emphasis on the density of inclusion bodies observed in colonic tissues. However, the interpretation of CMV inclusion bodies in haematoxylin and eosin or immunohistochemistry staining can be influenced by the biopsy location, such as at the ulcer margin or base, which may introduce bias [19]. Additionally, counting the number of inclusion bodies is a time-consuming process for pathologists, and there is currently no standardized cut-off to define high-grade disease. Considering the therapeutic regimen and duration, the preferred antiviral agent is intravenous ganciclovir at a dose of 5–7.5 mg/kg twice daily for 2 weeks [4, 20, 21].

In general, hospitalised patients with IBD tend to exhibit more severe inflammatory activity and more complicated disease than outpatients. Cytomegalovirus infection in hospitalised patients with IBD not only exacerbates gastrointestinal symptoms but also increases the risk of poor outcomes [7]. Therefore, maintaining a high level of clinical suspicion of CMV infection and implementing antiviral treatment are crucial for reducing the risk of colitis relapse in this patient population, as recommended by previous studies [23]. Although there are various diagnostic tools for CMV colitis, immunohistochemistry staining of colonic tissue is essential and considered the gold standard for diagnosis [22, 24]. Limited research has been conducted on the clinical manifestations, risk factors, treatment options, outcomes, and prognostic factors of CMV colitis, as confirmed by IHC staining in hospitalised patients with IBD. However, the potential benefits of antiviral treatments for this condition are not well understood. In this study, we investigated and addressed these issues.

## Materials and methods

### Patients

In this retrospective cohort study, we reviewed the medical records of eligible patients with IBD who were admitted to the Linkou Chang Gung Memorial Hospital between January 2000 and May 2021. Our study enrolled IBD inpatients admitted for acute flare-ups and management of related complications, with all participants undergoing IHC staining for CMV on colonic tissue samples. Outpatients and individuals without CMV IHC result on colonic tissues were excluded. Patients without colonic CMV IHC staining results were excluded. The diagnosis of CMV colitis was confirmed by positive CMV IHC staining of the colonic tissue and CMV-related tissue damage, with or without obvious viral inclusion bodies, using haematoxylin and eosin staining[25]. The patients were then divided into two groups (CMV and non-CMV groups) based on their colonic CMV IHC staining results. Monoclonal antibodies against the CMV pp65 antigen (Novocastra lyophilised mouse monoclonal antibody; Leica Microsystems, Wetzlar, Germany) were used for the CMV IHC staining. In consideration of treatment of CMV colitis, European Crohn’s and Colitis Organization (ECCO) guidelines recommend intravenous ganciclovir at a dose of 5 mg/kg twice daily for 5–10 days, followed by valganciclovir 900 mg daily until completion of a 2–3 week course, as the treatment of choice [22]. Therefore, we defined adequate antiviral treatment as the administration of intravenous ganciclovir and/or oral valganciclovir for a minimum of two weeks. In our research, we define ‘biologics failure’ as the patients had experienced loss of response to one or more biological treatments. Conversely, we refer to ‘biologics users’ as individuals presently receiving biologic therapy.

## Data collection

We collected demographic and clinical data, including age, sex, body mass index (BMI), underlying diseases (Crohn’s disease [CD], UC, diabetes mellitus, liver cirrhosis, coronary artery disease, end stage renal disease, other autoimmune diseases, and cancer), duration of IBD, baseline IBD complications (such as stricture, perforation, abscess, fistula, colon cancer, and previous IBD surgery), baseline medication, diagnostic date of CMV colitis, clinical presentations, antiviral treatment, outcomes (such as hospitalization times during follow-up, clinical remission, steroid free clinical remission, steroid dose changes, Crohn’s disease activity index (CDAI) change, Mayo score change, BMI change, occurrence of stricture, perforation, abscess, fistula, colon cancer, IBD surgery, overall IBD complications, recurrence, and death), and last follow-up date. We also collected laboratory data, including total white blood cell count (WBC), platelet (PLT), haemoglobin (Hb), creatinine (Cr), alanine aminotransferase (ALT), total bilirubin (Bil), albumin (ALB), and C-reactive protein (CRP) levels, CMV pp65 antigenemia, CMV viraemia (Light-Mix® Kit human cytomegalovirus [TIB Molbiol, Berlin, Germany, cut-off: Cp 35, 226 bp segment on glycoprotein B gene], COBAS® AmpliPrep/COBAS® TaqMan® CMV Test [Roche Diagnostics, Branchburg, NJ, USA, cut-off:150 copies/mL]), CMV serology, and Epstein-Barr virus (EBV) serology.

### Statistical analyses

Numerical data are reported as median (range), whereas categorical data are presented as absolute numbers and percentages. Continuous variables were compared using Mann-Whitney U tests, whereas categorical variables were analysed using χ2 and Fisher’s exact tests. Logistic regression models were used to determine independent risk factors for overall IBD complications. The variables with a *p*-value of less than 0.05 in the univariate analysis were considered for inclusion in the multivariate analysis. Statistical significance was set at *p* < 0.05, and the results are reported as odds ratios (ORs), 95% confidence intervals (CIs), or *p* values. All statistical analyses were performed using SPSS version 22.0 (IBM Corp., Armonk, NY).

## Results

A total of 158 patients with IBD were enrolled in this study. However, 40 patients were excluded, owing to a lack of colonic IHC staining results, resulting in the inclusion of 118 hospitalised patients with IBD, with 42 and 76 patients in the CMV and non-CMV groups, respectively. Among the enrolled patients, 49 had CD, and 69 had UC. The prevalence of CMV colitis in patients with IBD, CD, and UC was 35.6%, 22.4%, and 44.9%, respectively. The median age of the participants was 41.6 (3.4, 83.4) years, and most of them were males (64.4%). The median follow-up period was 21 months. There were no statistically significant differences in age, sex, BMI, underlying diseases (such as diabetes mellitus, liver cirrhosis, coronary artery disease, end-stage renal disease, other autoimmune diseases, and malignancy), IBD duration, or baseline IBD complications between the two groups.

Regarding baseline medications, some differences were observed between the CMV and non-CMV groups. The CMV group had a higher percentage of antibiotic exposure for both IBD (47.6% vs. 27.6%, *p* = 0.029) and CD (63.6% vs. 23.7%, *p* = 0.025). Additionally, subgroup analysis of UC revealed a higher percentage of hydrocortisone enema use in the CMV group (19.4% vs. 2.6%, *p* = 0.040). However, the use of oral steroids, immunomodulators, and biologics was not significantly different between the two groups. The biologic agents used in the CMV and non-CMV groups were infliximab (2.4% vs. 1.3%), adalimumab (4.8% vs. 11.8%), vedolizumab (14.3% vs. 13.2%), and ustekinumab (2.4% vs. 1.3%).

The most frequent symptoms of CMV colitis observed in patients were bloody stools (76.2%), diarrhoea (69.0%), and abdominal pain (61.9%). The CMV group had significantly lower albumin levels (*p* = 0.008) than the non-CMV group, particularly among patients with UC (*p* = 0.006). Other laboratory data, including WBC, lymphocyte, neutrophil, Hb, PLT, CRP, Cr, ALT, and Bil levels were not statistically different between the two groups. In terms of virology tests in the CMV group, the positivity rates for CMV IgM, CMV IgG, viraemia, and antigenemia were 12.9%, 96.6%, 45.5%, and 15.4%, respectively. The prevalence of CMV IgG positivity (96.6% vs. 72.7%, *p* = 0.08) and viraemia (45.5% vs. 0%, *p* = 0.03) were significantly higher in the CMV group than that in the non-CMV group. Additionally, the positivity rates for EBV viral capsid antigen (VCA) IgM, IgG, and viraemia were 0%, 100%, and 18.2%, respectively, in the CMV group. The EBV-VCA IgM (0% vs. 0%), IgG (100% vs. 92.9%, *p* = 0.568), and viraemia (18.2% vs. 13.6%, *p* = 1) levels were similar between the CMV and non-CMV groups.

Among the IBD patients with CMV infection, 79% received antiviral treatment, and 57% received sufficient treatment for at least 14 days. The recurrence rate was 23.1%, with a median duration of 25.4 months (range, 1.8–198 months) after antiviral treatment. No significant differences in clinical outcomes were observed between the CMV and non-CMV groups. Upon a thorough examination of the original data, it was observed that all surgical interventions for UC patients consisted solely of colectomies. Consequently, the incidence of surgeries in UC patients directly corresponds to the colectomy rates. As detailed in Table 1, the colectomy rates for UC patients were 35.5% in the CMV-infected group and 15.8% in the non-CMV group, demonstrating a notable trend towards significance (*p* = 0.059). Regarding CD patients, disease progression rates—assessed via the CDAI—were approximately 18.18% in the CMV group, in contrast to 15% in the non-CMV cohort, indicating no significant difference (*p* = 1.000). Furthermore, detailed insights into the progression rates among CD patients have been added to the results section for a more comprehensive understanding. Further details are presented in Table 1.

**Table 1:** Baseline characteristics and outcomes of CMV and non-CMV groups

Characteristics	IBD (*n* = 118)	CMV(*n* = 42)	Control (*n* = 76)	*P*		CD (*n* = 49)	CMV (*n* = 11)	Control (*n* = 38)	*P*		UC (*n* = 69)	CMV (*n* = 31)	Control (*n* = 38)	*P*
Age (years)	41.6 (3.4, 83.4)	45.1 (3.4, 83.4)	39.5 (15.6, 75.6)	0.116		37.6 (3.4, 75.6)	38 (3.4, 70.5)	36.6 (20.2, 75.6)	0.981		42 (13.5, 83.4)	46.1 (13.5, 83.4)	40.3 (15.6, 74.2)	0.070
Gender (Men)	76 (64.4%)	29 (69%)	47 (61.8%)	0.434		37 (75.5%)	8 (72.7%)	29 (76.3%)	1.000		39 (56.5%)	21 (67.7%)	18 (47.4%)	0.089
BMI	21.5 (13.6, 31.3)	20.3 (13.6, 29.8)	22.1 (14.1, 31.3)	0.182		21.5 (14.1, 31.3)	21.3 (16.6, 29.8)	21.5 (14.1, 31.3)	0.540		21.4 (13.6, 29.9)	20.2 (13.6, 26.3)	22.5 (14.5, 29.9)	0.166
Baseline IBD characteristics														
Disease duration (years)	4.0 (1.3, 22.5)	5.9 (1.3, 21.5)	3.5 (1.8, 22.5)	0.015*		3.7 (1.3, 22.5)	3.7 (1.3, 12.6)	3.6 (1.8, 22.5)	0.816		4.8 (1.3, 21.5)	7.6 (1.3, 21.5)	3.3 (2.0, 21.5)	0.005*
Montreal classification														
B1						24 (49.0%)	4 (36.4%)	20 (52.6%)	0.342					
B2						21 (42.9%)	4 (36.4%)	17 (44.7%)	0.737					
B3						8 (16.3%)	3 (27.3%)	5 (13.2%)	0.355					
L1						16 (32.7%)	5 (45.5%)	11 (28.9%)	0.466					
L2						4 (8.2%)	1 (9.1%)	3 (7.9%)	1.000					
L3						26 (53.1%)	4 (36.4%)	22 (57.9%)	0.208					
L4						28 (57.1%)	8 (72.7%)	20 (52.6%)	0.311					
CDAI						252.5 (46.9, 464.6)	268.5 (201, 379)	244.1 (46.9, 464.6)	0.442					
Mayo score											10 (5, 12)	10 (7, 12)	10 (5, 12)	0.838
Severity: EMS or SES-CD						7 (3, 24)	9 (3, 22)	6 (3, 24)	0.152		3 (1, 3)	3 (1, 3)	3 (1, 3)	0.999
Colonic stricture		13 (31.0%)					6 (54.5%)					7 (22.6%)		
IBD complications	47 (39.8%)	17 (40.5%)	30 (39.5%)	0.915		31 (63.3%)	10 (90.9%)	21 (55.3%)	0.038*		16 (23.2%)	7 (22.6%)	9 (23.7%)	0.914
The extent of CMV colitis														
Ascending		14 (33.3%)					3 (27.3%)					11 (35.5%)		
Transverse		17 (40.5%)					4 (36.4%)					13 (41.9%)		
Descending		27 (64.3%)					1 (9.1%)					26 (83.9%)		
Sigmoid		35 (83.3%)					4 (36.4%)					31 (100%)		
Rectum		33 (78.6%)					3 (27.3%)					30 (96.8%)		
IBD Medication														
Biological failure	33 (28%)	10 (23.8%)	23 (30.3%)	0.455		18 (36.7%)	2 (18.2%)	16 (42.1%)	0.178		15 (21.7%)	8 (25.8%)	7 (18.4%)	0.459
Biologics user	34 (28.8%)	11 (26.2%)	23 (30.3%)	0.847		15 (30.6%)	4 (36.4%)	11 (28.9%)	0.496		19 (27.5%)	7 (22.6%)	12 (31.6%)	0.367
5-ASA (oral)	8 (6.8%)	4 (9.5%)	4 (5.3%)	0.453		3 (6.1%)	3 (27.3%)	0 (0%)	0.009		5 (7.2%)	1 (3.2%)	4 (10.5%)	0.370
Oral prednisolone	64 (54.2%)	23 (54.8%)	41 (53.9%)	0.932		22 (44.9%)	6 (54.5%)	16 (42.1%)	0.510		42 (60.9%)	17 (54.8%)	25 (65.8%)	0.354
Dosage (mg/day)	5 (0, 45)	7.5 (0, 24)	5 (0, 45)	0.962		0 (0, 45)	10 (0, 24)	0 (0, 45)	0.350		10 (0, 30)	5 (0, 20)	10 (0, 30)	0.314
Azathiopurine	33 (28%)	10 (23.8%)	23 (30.3%)	0.455		17 (34.7%)	4 (36.4%)	13 (34.2%)	1.000		16 (23.2%)	6 (19.4%)	10 (26.3%)	0.496
Antibiotics	41 (34.7%)	20 (47.6%)	21 (27.6%)	0.029*		16 (32.7%)	7 (63.6%)	9 (23.7%)	0.025*		25 (36.2%)	13 (41.9%)	12 (31.6%)	0.373
Hydrocortisone enema	9 (7.6%)	6 (14.3%)	3 (3.9%)	0.067		2 (4.1%)	0 (0%)	2 (5.3%)	1.000		7 (10.1%)	6 (19.4%)	1 (2.6%)	0.040*
Clinical presentation														
Bloody stool	80 (67.8%)	32 (76.2%)	48 (63.2%)	0.147		24 (49%)	7 (63.6%)	17 (44.7%)	0.269		56 (81.2%)	25 (80.6%)	31 (81.6%)	0.921
Diarrhoea	75 (63.6%)	29 (69.0%)	46 (60.5%)	0.357		25 (51%)	6 (54.5%)	19 (50%)	0.791		50 (72.5%)	23 (74.2%)	27 (71.1%)	0.771
Pain	74 (62.7%)	26 (61.9%)	48 (63.2%)	0.893		34 (69.4%)	7 (63.6%)	27 (71.1%)	0.716		40 (58%)	19 (61.3%)	21 (55.3%)	0.614
Fever	18 (15.3%)	8 (19.0%)	10 (13.2%)	0.394		8 (16.3%)	3 (27.3%)	5 (13.2%)	0.355		10 (14.5%)	5 (16.1%)	5 (13.2%)	0.745
Laboratory data														
WBC (1000/µL)	8 (2.1, 26.5)	7.5 (4.2, 20.4)	8.1 (2.1, 26.5)	0.766		7.3 (2.1, 26.5)	7 (5.2, 20.4)	7.9 (2.1, 26.5)	0.792		8.7 (4, 20)	8.7 (4.2, 13.3)	8.7 (4, 20)	0.466
Haemoglobin (g/L)	119 (49, 159)	115 (74, 159)	121 (49, 156)	0.396		115 (72, 159)	112 (82, 159)	116 (72, 155)	0.590		121 (49, 156)	118 (74, 150)	125 (49, 156)	0.326
CRP (g/L)	0.09 (0.002, 1.765)	0.07 (0.002, 1.121)	0.09 (0.002, 1.765)	0.779		0.100 (0.002, 1.378)	0.110 (0.003, 1.121)	0.100 (0.002, 1.378)	0.915		0.06 (0.002, 176.5)	0.06 (0.002, 0.97)	0.07 (0.007, 1.765)	0.989
Albumin (g/L)	37 (14, 48)	30 (14, 4.4)	39 (2.2, 4.8)	0.008*		39 (22, 47)	42 (22, 44)	38 (25, 47)	0.842		36 (14, 48)	30 (14, 43)	40 (22, 48)	0.006*
CMV-IgM	6 (6.8%)	4 (12.9%)	2 (3.5%)	0.179		2 (5.7%)	1 (12.5%)	1 (3.7%)	0.410		4 (7.5%)	3 (13%)	1 (3.3%)	0.305
CMV-IgG	68 (81%)	28 (96.6%)	40 (72.7%)	0.008*		26 (76.5%)	7 (87.5%)	19 (73.1%)	0.645		42 (84%)	21 (100%)	21 (72.4%)	0.015*
CMV viremia	5 (16.7%)	5 (45.5%)	0 (0%)	0.003^*^		2 (15.4%)	2 (66.7%)	0 (0%)	0.038*		3 (17.6%)	3 (37.5%)	0 (0%)	0.082
CMV antigenemia	2 (5.4%)	2 (15.4%)	0 (0%)	0.117		0 (0%)	0 (0%)	0 (0%)	-		2 (8.7%)	2 (20.0%)	0 (0%)	0.178
Anti-viral agents														
Intravenous ganciclovir	18 (43.9%)	18 (43.9%)	-	-		4 (40%)	4 (40%)	-	-		14 (45.2%)	14 (45.2%)	-	-
Intravenous duration (days)	15 (7, 49)	15 (7, 49)	-	-		15 (7, 21)	15 (7, 21)	-	-		15 (8, 49)	15 (8, 49)	-	-
Oral Valganciclovir	26 (63.4%)	26 (63.4%)	-	-		5 (50%)	5 (50%)	-	-		21 (67.7%)	21 (67.7%)	-	-
Oral duration (days)	16 (6, 129)	16 (6, 129)	-	-		14 (7, 49)	14 (7, 49)	-	-		16 (6, 129)	16 (6, 129)	-	-
Total duration (days)	22 (7, 129)	22 (7, 129)	-	-		21 (7, 64)	21 (7, 64)	-	-		23.5 (7, 129)	23.5 (7, 129)	-	-
Outcomes														
Recurrence	6 (23.1%)	6 (23.1%)	-	-		2 (33.3%)	2 (33.3%)	-	-		4 (20%)	4 (20.0%)	-	-
Recurrence time (months)	25.4 (1.8, 198)	25.4 (1.8, 198)	-	-		68.9 (1.8, 136)	68.9 (1.8, 136)	-	-		25.4 (7.4, 198)	25.4 (7.4, 198)	-	-
Clinical remission	81 (68.6%)	27 (64.3%)	54 (71.1%)	0.448		36 (73.5%)	8 (72.7%)	28 (73.7%)	1.000		45 (65.2%)	19 (61.3%)	26 (68.4%)	0.536
Steroid free clinical remission	65 (55.1%)	22 (52.4%)	43 (56.6%)	0.661		28 (57.1%)	6 (54.5%)	22 (57.9%)	1.000		37 (53.6%)	16 (51.6%)	21 (55.3%)	0.762
Hospitalization times	2 (0, 27)	2 (0, 27)	2 (0, 12)	0.180		3 (0, 27)	2 (0, 27)	3 (0, 12)	0.561		2 (0, 12)	2 (0, 9)	2 (0, 12)	0.434
Steroid dose change	-4.5 (-44, 1)	-7 (-24, 1)	-4 (-44, 1)	0.938		0 (-44, 1)	-9 (-24, 1)	0 (-44, 1)	0.358		-9 (-30, 1)	-5 (-20, 1)	-9 (-30, 1)	0.223
CDAI change	-116.3 (-396.6, 88)	-155 (-322, 54)	-115.9 (-396.6, 88)	0.896		-115.4 (-396.6, 88)	-162.8 (-322, -68)	-112.6 (-396.6, 88)	0.533		-122.6 (-183, 54)	54 (54, 54)	-124.4 (-183, -120.7)	0.500
Mayo score change	-6 (-12, 1)	-5 (-12, 1)	-6 (-11, 0)	0.929		-	-	-	-		-6 (-12, 1)	-5 (-12, 1)	-6 (-11, 0)	0.929
BMI change	0.8 (-5.5, 13.3)	0.6 (-3.6, 5.3)	1 (-5.5, 13.3)	0.584		0.8 (-3.3, 13.3)	1.4 (-0.9, 3.8)	0.8 (-3.3, 13.3)	0.828		0.9 (-5.5, 7.5)	0.1 (-3.6, 5.3)	1.1 (-5.5, 7.5)	0.447
IBD complications	53 (44.9%)	20 (47.6%)	33 (43.4%)	0.661		31 (63.3%)	7 (63.6%)	24 (63.2%)	1.000		22 (31.9%)	13 (41.9%)	9 (23.7%)	0.106
Stricture	38 (32.2%)	12 (28.6%)	26 (34.2%)	0.530		25 (51.0%)	5 (45.5%)	20 (52.6)	0.675		13 (18.8%)	7 (22.6%)	6 (15.8%)	0.473
Perforation	6 (5.1%)	3 (7.1%)	3 (3.9%)	0.665		4 (8.2%)	2 (18.2%)	2 (5.3%)	0.214		2 (2.9%)	1 (3.2%)	1 (2.6%)	1.000
Abscess	9 (7.6%)	5 (11.9%)	4 (5.3%)	0.277		5 (10.2%)	2 (18.2%)	3 (7.9%)	0.311		4 (5.8%)	3 (9.7%)	1 (2.6%)	0.319
Fistula	13 (11.0%)	3 (7.1%)	10 (13.2%)	0.375		11 (22.4%)	1 (9.1%)	10 (26.3%)	0.415		2 (2.9%)	2 (6.5%)	0 (0%)	0.198
Colon cancer	4 (3.4%)	3 (7.1%)	1 (1.3%)	0.128		1 (2%)	1 (9.1%)	0 (0%)	0.224		3 (4.3%)	2 (6.5%)	1 (2.6%)	0.584
IBD surgery	36 (30.5%)	16 (38.1%)	20 (26.3%)	0.183		19 (38.8%)	5 (45.5%)	14 (36.8%)	0.729		17 (24.6%)	11 (35.5%)	6 (15.8%)	0.059
Death	5 (4.6%)	2 (5.9%)	3 (4.0%)	0.646		2 (4.1%)	0 (0%)	2 (5.3%)	1.000		3 (5%)	2 (8.7%)	1 (2.7%)	0.552
Follow-up duration (months)	21.1 (0, 192.1)	21.3 (0.4, 192.1)	21.1 (0, 57.6)	0.467		22.8 (0.3, 63.3)	26.3 (10.4, 63.3)	21.8 (0.3, 57.6)	0.465		19.1 (0, 192.1)	19.1 (0.4, 192.1)	18.5 (0, 56.1)	0.546

Overall, the IBD complications included strictures, abscesses, fistulas, colon cancer, and IBD-related surgeries. Abbreviations:5-ASA, 5-aminosalicylic acid; BMI, body mass index; CDAI, Crohn’s disease activity index; CI, confidence interval; CMV, cytomegalovirus; CRP, C-reactive protein; IBD, inflammatory bowel disease; UC, ulcerative colitis; WBC, white blood cell; EMS, endoscopic mayo subscore; SES-CD, Simple Endoscopic Score for Crohn’s Disease *p < 0.05

**Fig. 1:**
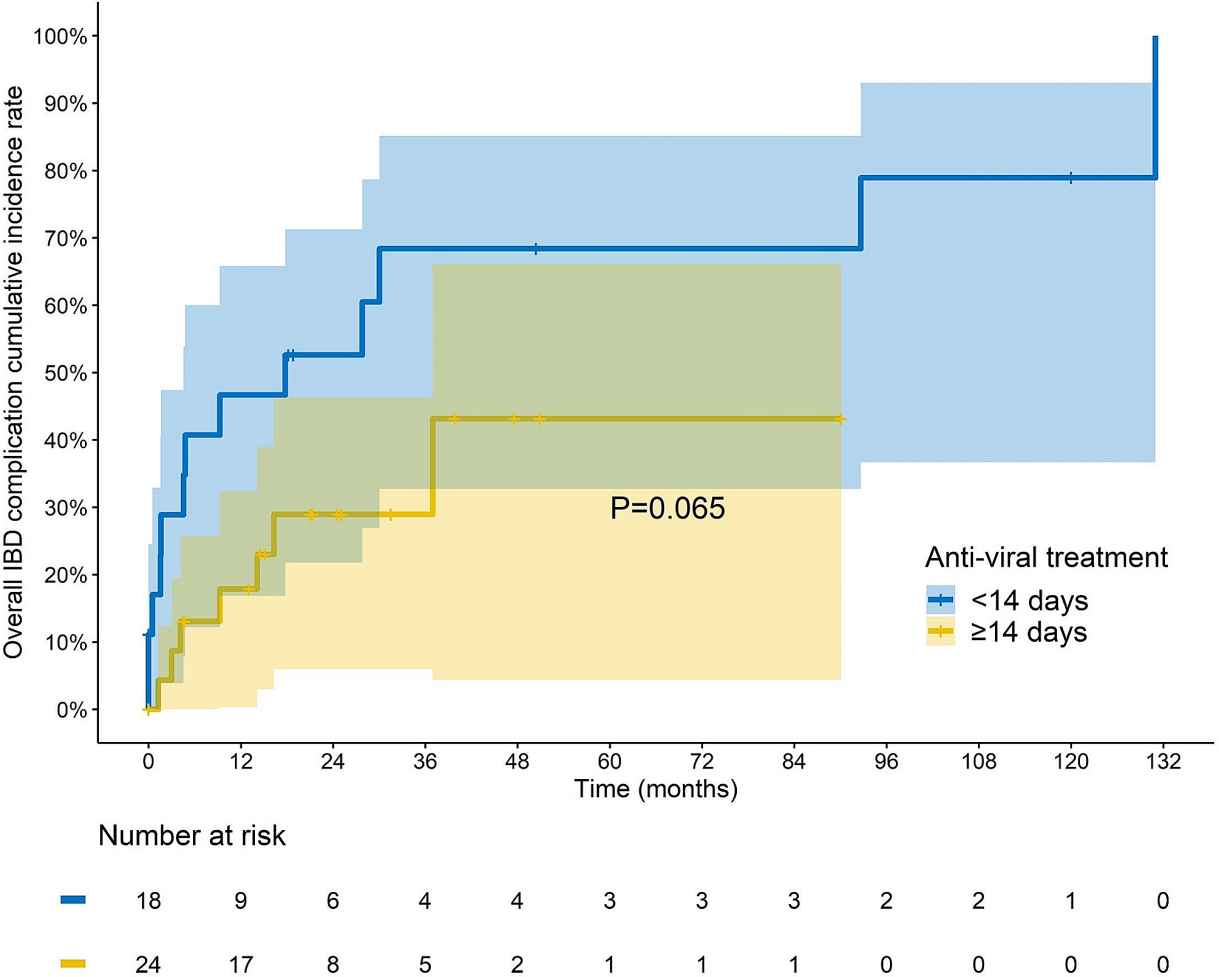
Kaplan-meier curve analysis of overall Inflammatory Bowel Disease (IBD) Complications in IBD Patients with Cytomegalovirus Colitis (CMV). IBD patients with CMV colitis who received adequate antiviral treatment ( ≧ 14 days) demonstrated a tendency towards reduced complication, compared to insufficient treatment (< 14 days) (log-rank *p* = 0.065)

Among the patients with IBD and CMV colitis, those who did not receive adequate antiviral treatment had significantly higher rates of overall complications (72.2% vs. 43.4%, *p* = 0.028) and surgery (55.6% vs. 26.3%, *p* = 0.017) than those in the non-CMV group. In contrast, adequate antiviral treatment led to a lower complication rate in the CMV group (29.2% vs. 72.2%, *p* = 0.006), particularly in the CD group (20% vs. 100%, *p* = 0.015) (Table 2). In the multivariate analysis, CMV colitis (OR, 3.532; 95% CI, 1.012–12.331, *p* = 0.048), biological failure (OR, 4.953; 95% CI, 1.91–12.842, *p* = 0.001), and adequate antiviral treatment (OR, 0.108; 95% CI, 0.023–0.512, *p* = 0.005) were identified as independent factors for predicting overall IBD complications (Table 3). Moreover, according to the Kaplan-Meier analysis, patients who received adequate antiviral treatment demonstrated a clear trend towards reduced complications, although the log-rank *p*-value was not statistically significant (*p* = 0.065) (Fig. 1).

**Table 2:** Impact of adequate antiviral treatment on overall IBD complications in CMV group

	IBD + CMV (*n* = 42)		*P*		CD + CMV (*n* = 11)		*P*		UC + CMV (*n* = 31)		*P*
Antiviral treatment	Adequate (*n* = 24)	Inadequate (*n* = 18)			Adequate (*n* = 5)	Inadequate (*n* = 6)			Adequate (*n* = 19)	Inadequate (*n* = 12)	
Overall IBD complications	7 (29.2%)	13 (72.2%)	0.006*		1 (20%)	6 (100%)	0.015*		6 (31.6%)	7 (58.3%)	0.141

Adequate antiviral treatment: intravenous ganciclovir and/or oral valganciclovir for at least 14 days. Overall, the IBD complications included strictures, abscesses, fistulas, colon cancer, and IBD-related surgeries. Abbreviations: CD, Crohn’s disease; CMV, cytomegalovirus; IBD, inflammatory bowel disease; UC, ulcerative colitis; WBC, white blood cell; * *P* < 0.05, calculated using the Chi-square or Fisher’s exact test

**Table 3:** Predictors of overall complications in hospitalized IBD patients

Characteristics	Univariable analysis				Multivariable analysis		
	OR	95% CI	*p*-value		OR	95% CI	*p*-value
Age (years)	0.994	(0.972–1.015)	0.564				
Gender (Male)	0.403	(0.182–0.893)	0.025*		0.449	(0.188–1.072)	0.071
BMI	1.039	(0.943–1.146)	0.441				
CMV colitis	1.185	(0.556–2.524)	0.661		3.532	(1.012–12.331)	0.048*
Laboratory data							
WBC	0.964	(0.873–1.063)	0.461				
Haemoglobin	0.924	(0.788–1.083)	0.329				
CRP	1.000	(0.99–1.01)	0.978				
Albumin	1.018	(0.535–1.937)	0.956				
IBD duration (months)	1.007	(0.999–1.015)	0.072		1.005	(0.997–1.013)	0.256
Clinical presentation							
Bloody stool	0.632	(0.291–1.374)	0.247				
Diarrhoea	0.499	(0.233–1.068)	0.073				
Pain	0.966	(0.456–2.044)	0.928				
Fever	0.978	(0.356–2.684)	0.965				
IBD Medication							
Biological failure	3.484	(1.492–8.134)	0.004*		4.953	(1.91–12.842)	0.001*
Biologics user	1.575	(0.707–3.509)	0.266				
5-ASA	2.153	(0.49–9.458)	0.310				
Oral prednisolone	0.684	(0.33–1.42)	0.308				
Azathioprine	0.869	(0.386–1.957)	0.735				
Antibiotics	0.592	(0.272–1.288)	0.186				
Antiviral treatment ≧ 14 days	0.613	(0.268–1.402)	0.247		0.108	(0.023–0.512)	0.005*

Overall, the IBD complications included strictures, abscesses, fistulas, colon cancer, and IBD-related surgeries. Abbreviations: ALT, Alanine aminotransferase; 5-ASA, 5-aminosalicylic acid; BMI, body mass index; CDAI, Crohn’s Disease Activity Index; CI, confidence interval; CMV, cytomegalovirus; CRP, C-reactive protein; IBD, inflammatory bowel disease; OR, odds ratio; WBC, white blood cell; *p < 0.05, calculated using logistic regression analysis

## Discussion

Cytomegalovirus colitis has been associated with a poor prognosis in IBD, including severe disease activity, surgery, and hospitalisation, particularly in patients with UC [26, 27]. However, the role of anti-viral therapy remains debatable, owing to heterogeneous study populations and diagnostic tools. According to the ECCO guidelines, antiviral therapy is recommended for steroid-refractory IBD patients with CMV colitis [22]. However, there is limited information on the role of antiviral treatment for CMV colitis in hospitalised patients with IBD. In this retrospective cohort study, we focused on hospitalised IBD patients with CMV IHC staining results and comprehensively analysed the clinical manifestations, risk factors, treatments, fifteen variable IBD outcomes, prognostic factors, and benefits of adequate antiviral treatment.

The prevalence of CMV colitis in patients with IBD varies, depending on the diagnostic tests used and study population. According to the ECCO guidelines, confirming active CMV colitis in IBD requires IHC, tissue polymerase chain reaction, or both, and should be the standard test [22]. In this study, the prevalence of CMV colitis in hospitalised patients with IBD, CD, and UC was found to be 35.6%, 22.4%, and 44.9%, respectively. This incidence was significantly higher than the previously reported incidence of 1.6% at the National Taiwan University Hospital, likely due to the inclusion of patients without colonic CMV histopathological examination and lack of IHC stain in every specimen [23]. Another German study also reported a higher incidence of 21% in hospitalized IBD patients who received tests for CMV infection [7]. In a systematic review, the incidence of CMV colitis in UC was noted to be 5.6 times higher than in CD [28], whereas in our study, the difference was only twice. The discrepancy observed could be linked to fewer colonoscopies being performed, leading to a decrease in biopsies for CMV IHC staining in patients with CD during acute flare-ups, as noted in previous studies. Therefore, the incidence rate of CMV colitis in inpatients with IBD has been underestimated in previous studies and in daily practice, especially in patients with CD. Furthermore, concurrent CMV colitis emerged as an independent factor for IBD-related complications, highlighting the critical role of adequate antiviral treatment in the management of hospitalized IBD patients.

In this study, we identified antibiotic exposure and hypoalbuminemia as the risk factors for CMV colitis in patients with IBD. Previous studies have shown that antibiotic exposure can increase the risk of CMV reactivation and gastrointestinal diseases due to dysbiosis [29–31]. Additionally, hypoalbuminemia, which is an indicator of poor nutritional status, has been previously reported to be associated with CMV diseases [33, 34]. Furthermore, the use of hydrocortisone enemas was found to be associated with an increased risk of CMV colitis in patients with UC. This may be related to potentially compromised mucosal immunity associated with corticosteroid use. The results of this study did not show a significant association between the use of oral glucocorticoids, azathioprine, or biologics and the risk of CMV colitis in patients with IBD. A meta-analysis of UC patients indicated that glucocorticoids, immunosuppressants, azathioprine, severe disease activity, pancolitis, and older age at UC onset may be associated with an increased risk of CMV reactivation [35]. However, it should be noted that our study population consisted of hospitalised IBD patients in a medical center, which may differ from studies that included all patients with UC. Additionally, our study utilised diagnostic criteria based on IHC for CMV colitis, whereas other studies focused on CMV reactivation. These differences in study populations and diagnostic criteria should be considered when interpreting our results.

It is crucial to distinguish between CMV infection and CMV disease, and the diagnosis or exclusion of CMV colitis should not be solely based on serology, antigen, or virus detection in the blood without evidence of CMV colonic tissue invasion. Previous studies have demonstrated poor correlation between these markers and active CMV gastrointestinal diseases, underscoring the need for accurate diagnostic methods, such as colonic tissue biopsy with IHC staining [24, 36]. In our study, based on the available data on CMV serology, antigen, and virus, the positive rates of CMV IgM, IgG, and viremia were 12.9%, 96.6%, and 45.5%, respectively. This suggests that most cases of CMV colitis are related to reactivation rather than primary infection, which is consistent with previous research [37]. The prevalence of CMV viremia (45.5%) in our study was found to be higher than that reported in the previously published studies (30%) [19]. Additionally, the rate of latent EBV infection was also high, with 94.7% of all patients and 100% of the CMV group testing positive for EBV-VCA IgG. This finding is noteworthy for physicians ruling out CMV and EBV infections in Taiwan. We conducted a univariable analysis to assess the impact of EBV infection on IBD-related complications, which yielded a *p*-value of 0.999, indicating no significant association. However, due to substantial missing data on EBV and the absence of tissue Epstein-Barr Encoded Small RNA In Situ Hybridization (EBER) staining to definitively confirm EBV colitis, we opted not to include these results in Table 3. The CMV and non-CMV groups demonstrated comparable IBD-related outcomes, primarily due to the fact that a substantial proportion of CMV colitis patients (79%) received antiviral therapy. Furthermore, when contrasting the 24 IBD patients with untreated CMV colitis against the non-CMV group, we observed a significantly higher incidence of overall IBD complications (72.2% vs. 43.4%, *p* = 0.028) and IBD-related colectomy rates (55.6% vs. 26.3%, *p* = 0.017). These findings align with the data presented in Table 3, indicating that CMV colitis acts as a progressive factor for overall IBD complications, while appropriate antiviral treatment serves as a protective factor against such complications.

The ECCO guidelines recommend testing for CMV colitis in patients with refractory IBD, particularly if they do not respond to immunosuppressive therapy [22]. Antiviral therapy is recommended for patients with steroid-refractory IBD with CMV colitis. Recent studies have suggested that antiviral therapy may be beneficial for UC patients with a high tissue CMV viral load [38, 39]. However, there is currently no standard cut-off point for defining high-grade CMV disease, and potential sampling bias and the time-consuming nature of counting inclusion bodies may limit its clinical application. With adequate antiviral treatment in CMV group, the rate of complications decreased significantly, especially in patients with CD. This suggests that timely diagnosis and appropriate antiviral treatment may improve outcomes in hospitalised patients with IBD and CMV colitis, particularly in those with CD.

In our study, recurrence is characterized by the resurgence of symptoms and signs accompanied by a positive IHC stain for CMV, following a period of clinical improvement and a previously negative IHC result after completing antiviral therapy. We observed a recurrence rate of 23.1%, which falls within the range reported by other studies (13.5–57%) [27, 32]. The variance in recurrence rates can be attributed to differences in study populations, treatment durations, and the criteria used to define recurrence.

In terms of predicting overall complications in IBD patients, our study found that CMV colitis (OR, 3.532; 95% CI, 1.012–12.331, *p* = 0.048), biological treatment failure (OR, 4.953; 95% CI, 1.91–12.842, *p* = 0.001), and receiving adequate antiviral treatment (OR, 0.108; 95% CI, 0.023–0.512, *p* = 0.005) were identified as independent factors. These findings suggest that in IBD inpatients with active disease, CMV colitis should be considered as a pathogen that can exacerbate inflammation rather than a mere by-stander. Biological failure is often indicative of a long refractory disease course, and is associated with a higher rate of complications. Our multivariate analysis demonstrated that adequate antiviral treatment decreased the overall complication rate (Table 3). Additionally, in the Kaplan-Meier analysis of overall complications, there was a noticeable trend towards reduced complications in patients who received sufficient antiviral treatment, although the sample size was not large enough to demonstrate statistical significance (log-rank *p* = 0.065).

It should be noted that this study has several limitations, including its retrospective design, single-center setting, and lack of inclusion body quantification. Further large-scale randomized controlled trials are warranted to validate our findings and confirm the potential benefits of antiviral treatment in this patient population.

## Conclusion

Our study revealed that CMV colitis is linked to a heightened risk of complications in hospitalised patients with IBD, and that appropriate antiviral treatment can lead to improved outcomes. Healthcare providers should maintain a high level of suspicion of CMV colitis in hospitalised patients with IBD and consider prompt and appropriate antiviral treatments.

## Data Availability

The datasets used and/or analysed in the current study are available from the corresponding author upon reasonable request.
